# Estimating the serial intervals of SARS‐CoV‐2 Omicron BA.4, BA.5, and BA.2.12.1 variants in Hong Kong

**DOI:** 10.1111/irv.13105

**Published:** 2023-02-08

**Authors:** Zihao Guo, Shi Zhao, Carrie Ho Kwan Yam, Conglu Li, Xiaoting Jiang, Tsz Yu Chow, Ka Chun Chong, Eng Kiong Yeoh

**Affiliations:** ^1^ JC School of Public Health and Primary Care, Faculty of Medicine Chinese University of Hong Kong Hong Kong China; ^2^ Centre for Health Systems and Policy Research, Faculty of Medicine Chinese University of Hong Kong Hong Kong China

**Keywords:** COVID‐19, disease transmission, Hong Kong, SARS‐CoV‐2 omicron variant, serial infection interval

## Abstract

Empirical evidence on the epidemiological characteristics of the emerged SARS‐CoV‐2 variants could shed light on the transmission potential of the virus and strategic outbreak control planning. In this study, by using contact tracing data collected during an Omicron‐predominant epidemic phase in Hong Kong, we estimated the mean serial interval of SARS‐CoV‐2 Omicron BA.4, BA.5, and BA.2.12.1 variants at 2.8 days (95% credible interval [CrI]: 1.5, 6.7), 2.7 days (95% CrI: 2.1, 3.6), and 4.4 days (95% CrI: 2.6, 7.5), respectively, with adjustment for right truncation and sampling bias. The short serial interval for the current circulating variant indicated that outbreak mitigations through contact tracing and case isolation would be quite challenging.

## 1 INTRODUCTION

As one of the genetic variants of concern (VOCs) of SARS‐CoV‐2 declared by the World Health Organization (WHO), the Omicron (B.1.1.529) variants spread at a rapid rate with novel genetic mutations persistently reported globally. Two emerging Omicron subvariants that were first detected in South Africa in January 2022, that is, BA.4 and BA.5, have risen public health concerns due to their transmission advantages against previous circulating variants.^1^, ^2^ Another subvariant of the Omicron lineage, BA.2.12.1 variants, emerged in the United States in February 2022 and also outcompeted the Omicron BA.2 locally, though it was subsequently replaced by BA.4 and BA.5.^3^

Current immunologic knowledge indicated that BA.4 and BA.5 variants had stronger resistance to the antibody elicited by vaccination or by previous infections of BA.1 or BA.2.^4^ However, little is known about the epidemiological characteristics of these variants, in particular, the serial interval (SI), which is defined as the time interval between the symptom onset date of primary and secondary cases. The knowledge of the distribution of SI is essential for informing the responsiveness of the control measures (i.e., contact tracing) and reliable estimation of key biological parameters. In this study, we used contact tracing data in Hong Kong to estimate the SI distributions of Omicron BA.4, BA.5, and BA.2.12.1 variants.

## 2 METHOD

### 2.1 Data

We collected line‐list contact tracing data of each individual SARS‐CoV‐2 infection reported from May 1 to July 17, 2022, from the Centre for Health Protection of the Department of Health in Hong Kong. We extracted the information of symptom onset date, case confirmation date, hospital admission date, contact tracing history, vaccine history, and genotype of the SARS‐CoV‐2 infected. To obtain SI observations, we constructed the infector–infectee transmission pairs of reported cases based on the contact tracing history.

### 2.2 Identification of transmission pairs

Based on the contact tracing history for cases with known symptom onset dates provided by the Centre for Health Protection of Hong Kong, we first identified case clusters, comprising a group of epidemiologically linked cases. Case clusters could involve one or multiple generations. Within the case clusters, cases marked by “imported” (cases that acquired infection outside Hong Kong based on the symptom onset dates and recent travel histories) or “local” (cases acquired infection locally and without recent travel history) were considered as the index cases (infector) of the cases in the secondary generation (marked by “close contact with local” or “close contact with imported”). For later generations where cases were all marked by “close contact with local” or “close contact with imported,” the infectors were determined only by the reported contact tracing history; that is, cases first exposed to the index cases or previous generations were the infector of the current generation. Infector–infectee transmission pairs were then resolved from case clusters. Infectees with two or more possible infectors were excluded from the analysis. Asymptomatic cases and unlink cases (i.e., cases that were associated with certain contact settings but were not epidemiologically linked with others and cases that were recorded linking to multiple infectors) were excluded from the analysis. We also excluded the pairs with SIs exceeding 15 days or below −5 days to ensure biologically plausible SI distributions.^5^

### 2.3 Statistical analysis

We denoted $S_{i}$ as the SI for the ith transmission pair, which was defined as the time interval between the illness onset date of the infector and that of his/her associated infectee. We assumed the SI of the BA.4, BA.5, and BA.2.12.1 followed a gamma distribution, denoted by $f\left(.\right)$. For observed negative SI (pre‐symptomatic transmission), we added a shift in $f\left(.\right)$. During the early phase of an outbreak, shorter SI is more likely to be identified due to the exponential growth of case numbers.^6^ We corrected such sampling bias in $f^{\prime }\left(.\right)$ by adjusting the exponential growth with rates *r* of 0.04, 0.02, and 0.04 per capita per day (estimated from the epidemic curve) for BA.2.12.1, BA.4, and BA.5, respectively. We also conducted sensitivity analysis using different exponential growth rates (from 0.01 to 0.06). The sampling‐bias‐adjusted distribution function $f^{\prime }\left(S_{i}\right)$ is given by^7^:

$$f^{\prime }\left(S_{i}\right)=\frac{f\left(S_{i}\right)e^{-rS_{i}}\;}{\int_{-\infty }^{\infty }f\left(S_{i}\right)e^{-rS_{i}}dS_{i}\;}.$$

Additionally, we also considered the right truncation of the time interval^8^; that is, the SI generated by each infector is truncated due to timely case isolation. Thus, the truncation‐adjusted distribution function is given by

$$f_{\text{adjust}}\left(S_{i}\right)=\frac{f^{\prime }\left(S_{i}\right)}{F^{\prime }\left(T_{i}\right)}.$$

Here, the $F^{\prime }\left(.\right)$ is the cumulative density function of $f^{\prime }\left(.\right)$. The $T_{i}$ is the confirmation delay, that is, the time interval between the symptom onset and isolation of the infector for the ith transmission pair. We used the hospital admission date as a surrogate of the isolation date. For cases without known admission dates, we used the case confirmation date instead. For a total of n transmission pairs identified for a certain type of Omicron subvariant, the likelihood function is given by

$$L_{\text{adjust}}=\prod_{i}^{n}f_{\text{adjust}}\left(S_{i}\right).$$

### 2.4 Parameter estimations

The parameters of gamma distribution were estimated by using the Metropolis–Hastings algorithm, which is a Markov chain Monte Carlo (MCMC) method, with noninformative prior distributions. The marginal posterior distribution was obtained from 100,000 iterations, among which the first 40,000 samples were discarded as for burn‐in. The convergence of each MCMC chain was checked by using the trace plot and Gelman–Rubin–Brooks convergence diagnostic. The median and the 95% credible interval (CrI) of the mean, standard deviation, median, and 95th percentile of the SI distributions were computed from the high‐density region of the 60,000 posterior samples.

## 3 RESULT AND DISCUSSION

**FIGURE 1:**
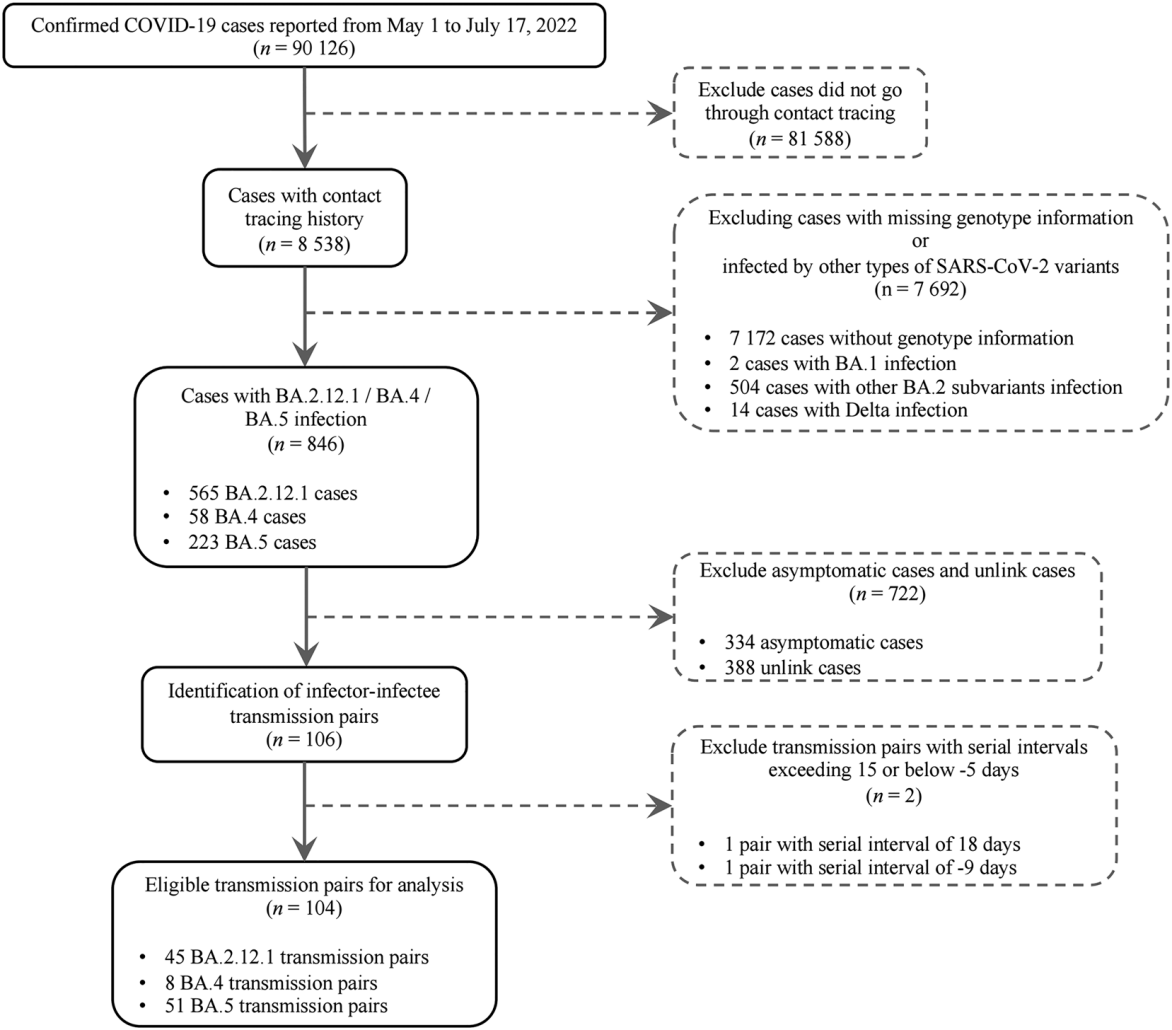
Flowchart for data collection.

**FIGURE 2:**
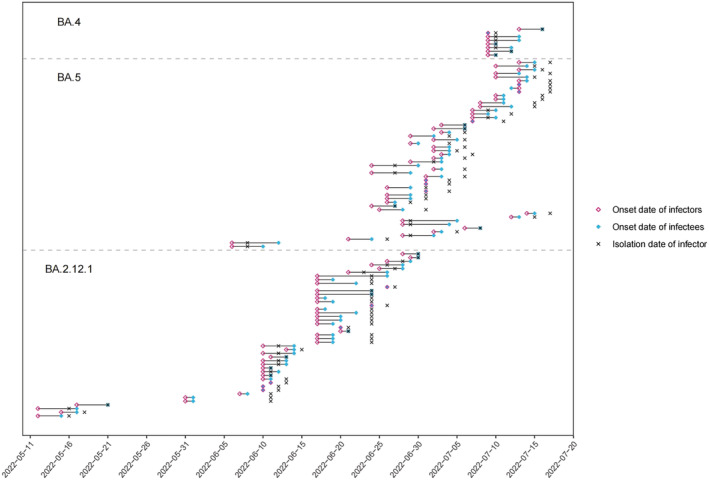
Identified transmission pairs for the Omicron BA.4, BA.5, and BA.2.12.1 variants, from May 1 to July 17, 2022. The edges between the colored points denoted the length of the serial interval.

**FIGURE 3:**
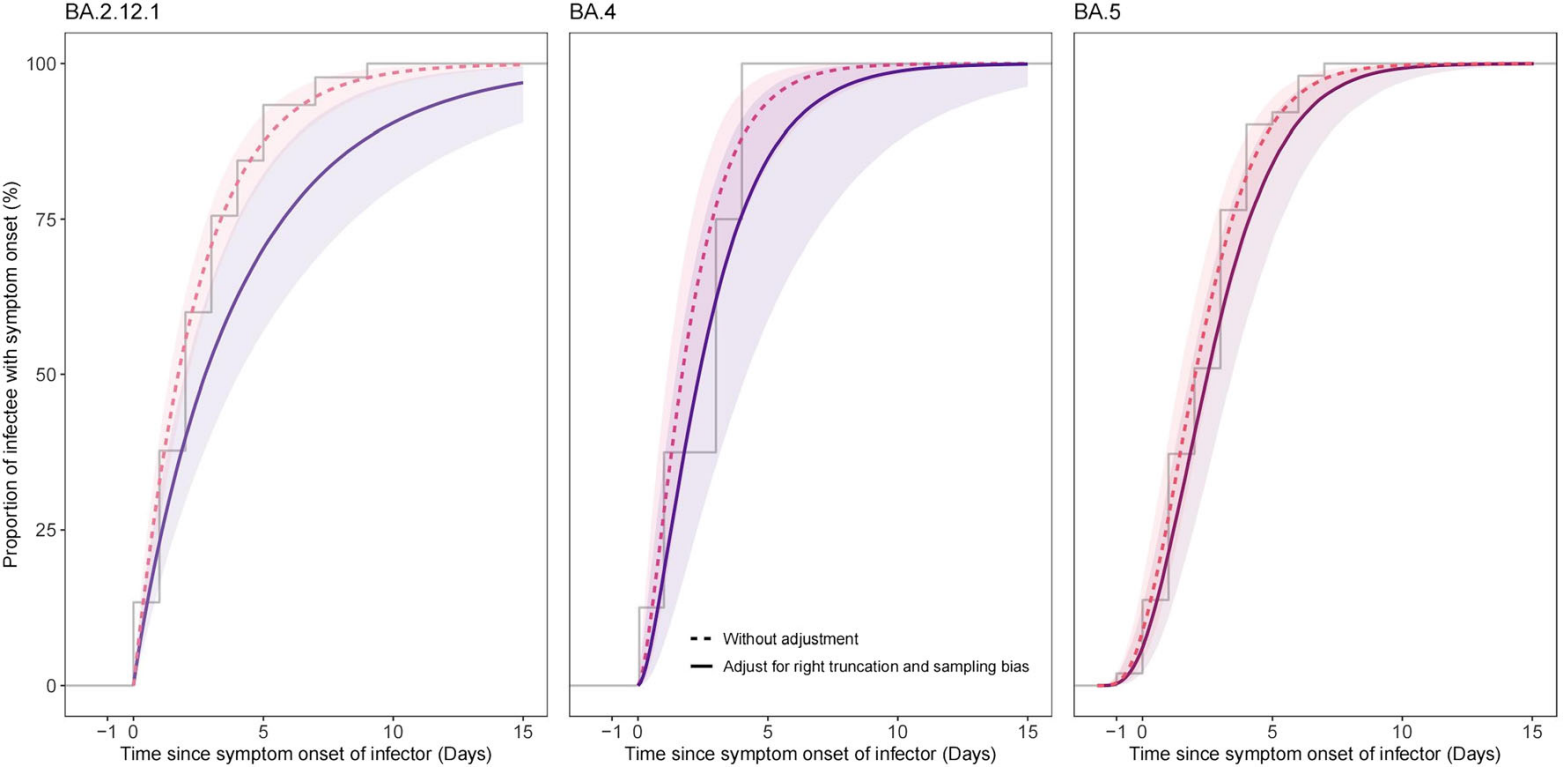
The empirical and estimated cumulative distribution functions of serial interval for Omicron BA.2.12.1, BA.4, and BA.5 variants. The step function represented the empirical cumulative density function. The curves represented the median, and the shaded area represented the 90% high‐density region of Markov chain Monte Carlo (MCMC) posterior samples.

The process of data collection was shown in Figure 1. A total of 90,126 confirmed cases were reported during the study period, of which 8538 went through contact tracings. Among the 8538 traced cases, 1366 cases had known genotype information, within which 846 cases were found infected by the Omicron BA.4/BA.5/BA.2.12.1 variants. After excluding asymptomatic cases and unlink cases, we identified a total of 104 transmission pairs including 8, 51, and 45 pairs with BA.4, BA.5, and BA.2.12.1 infections, respectively (Figures 1 and 2). Of all identified transmission pairs, there were 8, 43, and 40 received at least two doses of the COVID‐19 vaccine, respectively. The demographic characteristics of cases and contact settings where infection occurred between transmission pairs were not statistically different between the study samples and overall cases during the study period (Tables S1 and S2). The empirical mean SI estimates without adjusting for truncation and sampling bias were estimated at 2.1 days (95% CrI: 1.3, 3.6), 2.4 days (95% CrI: 1.9, 2.9), and 2.4 days (95% CrI: 1.8, 3.3) for BA.4, BA.5, and BA.2.12.1, respectively (Table 1 and Figure 3). During an ongoing epidemic, both exponential growth of case numbers and rapid case isolation measures could lead to an underestimation of the SI.^6^, ^8^, ^9^ We thus considered such bias in the analysis and the adjusted estimates approaches the intrinsic distribution of SI.^10^ After adjustments, the mean SI estimates were 2.8 days (95% CrI: 1.5, 6.7), 2.7 days (95% CrI: 2.1, 3.6), and 4.4 days (95% CrI: 2.6, 7.5) for BA.4, BA.5, and BA.2.12.1, respectively (Tables 1 and S3 and Figure 3). For BA.4, BA.5, and BA.2.12.1, the estimated 95th percentiles of SI were 7.1, 6.1, and 12.8 days, respectively (Table 1). Moreover, our model appeared to be less sensitive to the exponential growth rate (Table S4).

**TABLE 1:** The summary of estimated mean, SD, median, and 95th percentile of serial interval distributions for different Omicron subvariants.

Omicron subvariants	Model adjustment	Mean (95% CrI)	SD (95% CrI)	Median (95% CrI)	95th percentile (95% CrI)
BA.2.12.1 (*n* = 45)	Without adjustment	2.4 (1.8, 3.3)	2.3 (1.7, 3.5)	1.7 (1.2, 2.3)	7.1 (5.4, 10.1)
	Adjust for sampling bias	2.6 (2.0, 3.8)	2.5 (1.8, 3.9)	1.9 (1.3, 2.6)	7.7 (5.6, 11.6)
	Adjust for sampling bias and right truncation	4.4 (2.6, 7.5)	4.3 (2.4, 7.5)	2.9 (1.7, 4.8)	12.8 (7.3, 21.7)
BA.4 (*n* = 8)	Without adjustment	2.1 (1.3, 3.6)	1.6 (1.0, 3.1)	1.7 (0.9, 2.9)	5.2 (3.3, 9.6)
	Adjust for sampling bias	2.2 (1.3, 3.8)	1.6 (1.1, 3.6)	1.7 (1.0, 3.1)	5.4 (3.4, 9.6)
	Adjust for sampling bias and right truncation	2.8 (1.5, 6.7)	2.1 (1.1, 5.4)	2.2 (1.1, 5.3)	7.1 (3.8, 16.9)
BA.5 (*n* = 51)	Without adjustment	2.4 (1.9, 2.9)	2.0 (1.6, 2.6)	2.2 (1.7, 2.7)	5.5 (4.7, 6.6)
	Adjust for sampling bias	2.5 (2.0, 3.1)	1.8 (1.5, 2.3)	2.3 (1.8, 2.8)	5.8 (4.9, 7.1)
	Adjust for sampling bias and right truncation	2.7 (2.1, 3.6)	2.5 (1.9, 3.2)	2.5 (1.9, 3.2)	6.1 (5.0, 8.9)

Abbreviation: CrI, credible interval.

The empirical mean SI estimate of BA.2.12.1 variants was largely in line with previous estimates of 2.7 days for BA.2 variants in Hong Kong^11^ but relatively low than 3.3 days in United Kingdom.^12^ Because the SI of Omicron BA.4 and BA.5 variants appeared shorter than those of BA.2 and other preceding strains (e.g., Delta and Alpha),^13^, ^14^ this might contribute to explaining the selection advantages of BA.4 and BA.5 in many countries globally.^1^ As a recent study indicated that vaccination for both case‐patients of a transmission pair may potentially increase the SI,^5^ it is possible that the intrinsic SI for BA.4 and BA.5 may be smaller than what we obtained given that majority of included transmission pairs were fully vaccinated (i.e., two‐dose vaccination).

Our study had some limitations. First, our estimations relied on contact tracing data, and thus, any recall bias from the reported cases and/or case under‐ascertainment would affect the accuracy of the identified transmission pairs, which may bias our results. Second, contact tracing of cases was limited during the surge of cases, and the local surveillance systems could only trace a relatively small proportion of cases, thus, the sample size of transmission pairs was relatively small. In addition, selection bias on the transmission pairs could occur during a growth phase of cases as infectors that had more recent symptom onset were more likely to be sampled for an infectee.^6^ Nonetheless, we corrected for such bias in our statistical models, and we believe our results were less subjected to this bias. Last, because our sample sizes were small, we could not elucidate the effect of demographic factors (i.e., age and sex), types of contact setting, and vaccinations on the SI distribution of the Omicron subvariants.

In conclusion, our analysis showed a shorter SI for the novel SARS‐CoV‐2 Omicron subvariants during the current BA.5 global predominance phases. Thus, outbreak mitigations that relied on contact tracing and case isolations were challenging. We highlighted the need to continuously monitor the epidemiological feature of emerging COVID‐19 variants for a better understanding of their transmission potential for planning targeted control strategies in time.

## AUTHOR CONTRIBUTIONS

Conceptualization: Zihao Guo, Shi Zhao, and Ka Chun Chong. Data curation: Carrie Ho Kwan Yam and Tsz Yu Chow. Methodology: Zihao Guo and Shi Zhao. Formal analysis: Zihao Guo. Visualization: Zihao Guo. Validation: Shi Zhao and Ka Chun Chong. Writing—original draft: Zihao Guo. Writing—review & editing: Zihao Guo, Shi Zhao, Eng Kiong Yeoh, Ka Chun Chong, Conglu Li, and Xiaoting Jiang. Resources: Ka Chun Chong and Eng Kiong Yeoh. Project administration: Ka Chun Chong and Eng Kiong Yeoh. Supervision: Ka Chun Chong and Eng Kiong Yeoh.

## CONFLICT OF INTEREST STATEMENT

All authors declared no conflict of interest.

### PEER REVIEW

The peer review history for this article is available at https://publons.com/publon/10.1111/irv.13105.

## Supporting information


**Table S1.** Demographic characteristics of the included COVID‐19 cases and all confirmed COVID‐19 cases during the study period.
**Table S2.** Contact settings of included transmission pairs and all transmission pairs identified during the study period.
**Table S3.** Estimated parameters of the gamma distributed serial interval by the Omicron subvariants. The estimates of serial intervals were adjusted for right truncation and sampling bias.
**Table S4.** Mean serial interval estimates by the Omicron subvariants and exponential growth rate. The estimates of serial intervals were adjusted for right truncation and sampling bias.

## Data Availability

The dataset is not public‐available because the data are owned by third parties. Access to these data and permission could be inquired through the Hospital Authority and Department of Health, Hong Kong SAR Government.
