# Supplementary material for: Estimating the serial intervals of SARS‐CoV‐2 Omicron BA.4, BA.5, and BA.2.12.1 variants in Hong Kong
Source: Influenza Other Respir Viruses. 2023 Feb 8;17(2):e13105. doi: 10.1111/irv.13105 (PMC9942273; doi:10.1111/irv.13105)
Supplement: Supplementary file 1 — Table S1. Demographic characteristics of the included COVID‐19 cases and all confirmed COVID‐19 cases during the study period. Table S2. Contact settings of included transmission pairs and all transmission pairs identified during the study period. Table S3. Estimated parameters of the gamma distributed serial interval by the Omicron subvariants. The estimates of serial intervals were adjusted for right truncation and sampling bias. Table S4. Mean serial interval estimates by the Omicron subvariants and exponential growth rate. The estimates of serial intervals were adjusted for right truncation and sampling bias. [file IRV-17-e13105-s001.docx]

**Supplementary materials**

**Table S1.** Demographic characteristics of the included COVID-19 cases and all confirmed COVID-19 cases during the study period.

| **Characteristics** | **Included cases (*n* = 208)** | **All cases (*n* = 90 126)** | ***p –* value** |
| --- | --- | --- | --- |
| **Age, mean (SD)** | 44 (19.9) | 43 (20.6) | 0.483^*^ |
| **Sex** | | | |
| **Male, *n* (column %)** | 97 (47%) | 43 002 (48%) | 0.708^#^ |
| **Female, *n* (column %)** | 111 (53%) | 46 668 (52%) |  |

^*^ Two Sample t-test comparison of means.

^#^ Chi-square test of the frequencies.

**Table S2.** Contact settings of included transmission pairs and all transmission pairs identified during the study period.

| **Contact setting** | **Included transmission pairs (*n* = 104)** | **All transmission pairs (*n* = 4 151)** | ***p –* value** |
| --- | --- | --- | --- |
| **Household, *n* (column %)** | 74 (71%) | 3 103 (75%) | 0.065^*^ |
| **Workplace, *n* (column %)** | 8 (8%) | 142 (3%) |  |
| **Social, *n* (column %)** | 22 (21%) | 906 (22%) |  |

^*^ Chi-square test of the frequencies.

**Table S3.** Estimated parameters of the gamma distributed serial interval by the Omicron subvariants. The estimates of serial intervals were adjusted for right truncation and sampling bias.

| **Parameters** | **BA.2.12.1 (*n* = 45)** | **BA.4 (*n* = 8)** | **BA.5 (*n* = 51)** |
| --- | --- | --- | --- |
|  | **Median of the posterior distribution (95%CrI)** | **Median of the posterior distribution (95%CrI)** | **Median of the posterior distribution (95%CrI)** |
| Shape | 0.94 (0.69, 1.32) | 1.68 (0.78, 3.15) | 3.74 (2.49, 6.44) |
| Rate | 0.21 (0.12, 0.43) | 0.59 (0.20, 1.35) | 0.84 (0.53, 1.34) |
| Shift* | - | - | 1.71 (2.80, 1.16) |

*The shift parameter was only estimated for the Omicron BA.5 variant as negative serial intervals were only observed for BA.5 transmission pairs.

**Table S4.** Mean serial interval estimates by the Omicron subvariants and exponential growth rate. The estimates of serial intervals were adjusted for right truncation and sampling bias.

| **Exponential  growth rate** | **BA.2.12.1 (*n* = 45)** | **BA.4 (*n* = 8)** | **BA.5 (*n* = 51)** |
| --- | --- | --- | --- |
|  | **Mean serial interval** **(95%CrI)** | **Mean serial interval (95%CrI)** | **Mean serial interval (95%CrI)** |
| 0.01 | 3.98 (2.41, 7.04) | 2.81 (1.46, 6.74) | 2.60 (2.04, 3.39) |
| 0.02 | 4.13 (2.49, 7.21) | 2.83 (1.45, 6.71) | 2.65 (2.08, 3.54) |
| 0.03 | 4.29 (2.57, 7.27) | 2.87 (1.48, 7.43) | 2.66 (2.10, 3.46) |
| 0.04 | 4.37 (2.56, 7.53) | 2.88 (1.51, 7.95) | 2.69 (2.14, 3.59) |
| 0.05 | 4.62 (2.54, 7.89) | 2.91 (1.49, 7.88) | 2.75 (2.17, 3.68) |
| 0.06 | 4.69 (2.78, 7.71) | 2.97 (1.53, 8.98) | 2.79 (2.18, 3.89) |
